# Letter to the Editor

**DOI:** 10.1007/s00062-026-01634-8

**Published:** 2026-03-06

**Authors:** Johannes Gerber

**Affiliations:** Neuroradiology, Faculty of Medicine and University Hospital Carl Gustav Carus, TUD Dresden University of Technology, Dresden, Germany

Dear Editors,

With great interest, we read the recent meta-analysis titled *“Intravenous Tirofiban for Preventing Acute In-Stent Thrombosis in Emergent Carotid Artery Stenting”* by Anderson Matheus Pereira da Silva and colleagues [1].

The authors included four studies in their meta-analysis [2–5]: two originating from China [2, 5], one from Spain [3], and one from Switzerland [4]. However, we noted an important inconsistency between the stated scope of the meta-analysis and the studies included.

References [2] and [5] evaluate the efficacy and safety of tirofiban prior to stenting for symptomatic intracranial atherosclerotic stenosis, with all patients pretreated with dual antiplatelet therapy (DAPT) for at least five days before the procedure. This clinical setting differs significantly from emergent extracranial carotid artery stenting for acute ischemic stroke due to tandem occlusion, which is presumably the focus of the meta-analysis based on its title.

In contrast, references [3] and [4] are aligned with the intended scope: they report on emergent extracranial carotid artery stenting in the setting of acute tandem occlusive lesions and investigate pharmacological strategies—namely intravenous tirofiban—to prevent acute in-stent thrombosis under emergency conditions.

By combining studies on semi-acute or elective intracranial stenting in pretreated patients with those on emergent extracranial stenting in acute ischemic stroke, the meta-analysis merges fundamentally different clinical scenarios. This conflict may limit the validity of its conclusions and reduce its clinical applicability to real-world acute stroke management.

We believe a more focused analysis, limited to homogeneous patient populations and clinical contexts, is essential to draw meaningful conclusions.

## Data Availability

No datasets were generated or analysed during the current study.
